# Supplementary material for: Phytochemical Characterization of a Tree Tomato (Solanum betaceum Cav.) Breeding Population Grown in the Inter-Andean Valley of Ecuador
Source: Plants (Basel). 2022 Jan 20;11(3):268. doi: 10.3390/plants11030268 (PMC8838755; doi:10.3390/plants11030268)
Supplement: Supplementary file 1 [file plants-11-00268-s001.zip › plants-1483411-supplementary.pdf]

Table S1. Physical and chemical data of the 90 segregants of the tree tomato breeding population.

Segregant	L	a	b	TA	SS	SAR	TI	TP*	TF*	TC*	TAC*	VC*	FRAP*	ABTS*
1	75.55	10.39	47.5	1.12	11.2	10.00	1.62	6.65	2.52	360.32	1.06	98.22	58.38	55.78
2	73.42	8.65	47.03	0.99	11.5	11.62	1.57	7.45	3.25	412.58	2.93	78.29	82.19	71.24
3	74.78	10.08	49.22	0.92	10.9	11.85	1.51	6.20	1.60	316.19	n.d.	163.09	62.60	54.78
4	58.78	12.05	32.19	1.26	11.8	9.37	1.73	9.65	2.47	454.95	74.02	289.54	84.98	88.82
5	50.49	19.11	20.82	1.63	11.3	6.93	1.98	8.66	1.64	384.92	107.92	364.59	74.72	69.73
6	35.20	12.63	7.56	1.11	11.9	10.72	1.65	12.28	3.20	342.15	240.49	251.53	119.7	131.79
7	49.97	17.9	21.01	1.49	12.0	8.05	1.89	10.55	2.87	445.43	116.52	397.62	90.75	89.74
8	70.63	14.4	53.76	0.86	12.1	14.07	1.56	8.85	2.23	442.12	5.54	219.71	82.02	82.32
9	59.64	14.83	31.08	1.16	12.2	10.52	1.69	8.3	2.22	318.46	57.00	274.78	78.81	82.23
10	53.61	12.35	25.62	1.41	11.9	8.44	1.83	11.63	5.67	345.18	93.87	243.22	144.52	100.28
11	55.78	13.22	21.33	1.18	11.8	10.00	1.68	9.39	4.39	265.52	94.09	231.60	94.88	89.36
12	72.38	12.33	47.90	1.45	11.5	7.93	1.85	7.29	2.19	283.69	7.94	294.59	70.18	54.15
13	51.69	16.70	20.15	1.63	12.6	7.73	2.02	9.98	2.44	406.08	119.5	374.90	98.02	78.07
14	51.75	9.52	22.52	1.23	11.4	9.27	1.69	11.76	4.03	459.26	99.19	324.89	115.36	95.05
15	56.72	14.31	26.69	1.60	11.4	7.13	1.96	10.04	2.90	279.87	76.83	428.16	102.13	73.42
16	73.91	8.90	48.53	1.47	10.6	7.21	1.83	6.36	2.50	225.60	4.25	176.62	73.39	51.05
17	72.28	11.08	49.89	1.25	12.1	9.68	1.73	7.11	1.77	243.47	6.02	174.84	82.19	54.42
18	70.48	11.21	48.13	1.28	12.1	9.45	1.75	6.47	2.15	241.37	3.01	164.88	81.24	49.51
19	73.44	9.89	48.98	1.09	12.5	11.47	1.66	7.23	1.67	241.92	4.71	168.23	79.34	78.27
20	71.00	7.03	41.35	1.12	11.6	10.36	1.64	5.11	1.54	284.27	5.84	143.25	52.43	61.72
21	72.14	9.68	49.15	1.06	12.6	11.89	1.65	8.19	2.25	367.21	4.92	184.82	93.53	90.80
22	69.90	14.81	55.99	0.76	12.9	16.97	1.61	8.79	1.96	460.72	5.18	184.74	94.24	88.77
23	74.71	9.17	48.63	1.38	11.7	8.48	1.80	8.05	2.60	359.89	3.82	176.53	84.07	83.89
24	73.35	11.32	51.56	1.32	12.1	9.17	1.78	8.54	1.98	338.49	3.51	299.58	87.25	79.43
25	73.24	9.68	52.98	1.42	11.8	8.31	1.84	6.67	2.32	406.00	6.54	174.73	72.63	78.16
26	72.35	10.85	51.19	1.63	11.9	7.30	2.00	8.59	2.70	386.22	4.56	329.71	95.50	76.55
27	70.80	11.73	53.68	1.26	12.1	9.60	1.74	8.62	2.41	348.82	5.92	311.13	88.78	71.66
28	74.84	9.58	49.97	1.37	11.7	8.54	1.80	7.01	1.67	230.96	3.51	247.99	70.37	55.13

Continuation Table S1.

Segregant	L	a	b	TA	SS	SAR	TI	TP*	TF*	TC*	TAC*	VC	FRAP*	ABTS*
29	72.35	11.33	51.04	1.00	12.0	12.00	1.60	7.28	2.45	460.35	5.30	134.87	76.95	66.75
30	73.26	10.09	48.42	0.95	11.7	12.32	1.57	8.59	2.77	363.74	3.83	176.50	99.02	80.30
31	77.35	6.74	45.70	1.44	11.2	7.78	1.83	6.19	2.81	311.59	4.73	213.15	61.67	49.76
32	73.38	11.08	47.05	1.01	11.6	11.49	1.58	6.53	2.52	225.79	4.64	226.40	75.32	55.06
33	68.11	11.43	47.58	1.44	12.0	8.33	1.86	5.66	2.59	297.24	6.87	196.47	65.43	49.71
34	70.34	9.58	41.20	1.40	12.0	8.57	1.83	6.46	2.97	235.64	4.95	139.79	72.38	57.19
35	65.63	10.09	37.07	1.24	11.5	9.27	1.70	8.96	5.29	221.82	39.31	158.28	101.29	85.80
36	73.54	9.22	46.05	0.68	11.5	16.91	1.53	7.89	4.53	280.82	5.10	134.84	96.83	76.98
37	67.38	7.99	44.88	0.81	11.9	14.69	1.54	8.09	4.53	214.78	10.96	107.45	93.03	73.78
38	72.76	6.51	39.64	1.19	10.6	8.91	1.64	8.93	4.74	202.67	3.11	86.65	106.12	74.71
39	72.53	7.43	42.01	1.08	11.2	10.37	1.60	6.65	2.88	238.76	6.15	107.40	89.29	65.39
40	74.23	10.10	50.38	0.84	11.2	13.33	1.51	7.68	3.40	337.88	1.86	89.98	100.02	68.72
41	71.85	8.62	46.99	0.93	11.2	12.04	1.53	8.53	3.48	260.42	5.42	99.15	102.98	73.10
42	69.33	8.98	46.39	1.08	11.7	10.83	1.62	10.53	6.70	385.19	3.55	97.46	113.53	89.04
43	68.96	6.30	39.03	1.09	11.2	10.28	1.60	7.54	3.97	253.86	4.80	93.28	75.64	66.00
44	70.40	8.90	40.54	1.02	11.4	11.18	1.58	9.40	4.89	288.91	12.25	176.44	120.79	86.90
45	76.16	4.86	41.08	1.10	10.9	9.91	1.60	7.39	4.26	185.59	4.22	213.11	94.39	84.07
46	75.81	6.91	42.36	1.32	11.6	8.79	1.76	7.50	4.37	162.14	3.14	141.50	96.70	82.89
47	36.30	13.64	4.19	0.97	11.9	12.27	1.58	13.42	6.40	219.43	223.61	256.38	169.61	125.65
48	33.54	13.69	3.65	1.10	12.0	10.91	1.65	12.23	4.69	157.28	237.28	349.65	158.16	119.99
49	47.55	9.79	11.83	0.93	11.6	12.47	1.55	10.41	5.26	270.11	133.97	153.08	139.10	90.17
50	71.36	9.59	44.56	0.99	11.6	11.72	1.58	8.86	4.25	180.72	4.57	138.06	117.62	72.06
51	61.62	4.44	38.59	1.27	8.5	6.69	1.60	6.03	3.38	79.30	37.81	159.47	229.31	160.68
52	72.34	9.65	49.55	0.97	9.0	9.28	1.43	6.50	2.30	94.25	35.10	131.54	251.30	156.63
53	47.55	6.01	34.72	1.32	9.0	6.82	1.66	8.83	3.15	76.06	47.81	193.05	322.29	205.23
54	68.52	8.17	43.56	0.90	9.1	10.11	1.41	6.23	2.55	78.53	47.46	104.68	218.36	147.83
55	68.03	9.20	44.06	1.05	8.5	8.10	1.45	7.55	2.86	128.14	68.09	158.7	283.61	173.12

Continuation Table S1.

Segregant	L	a	B	TA	SS	SAR	TI	TP*	TF*	TC*	TAC*	VC	FRAP*	ABTS*
56	65.57	10.89	46.96	0.97	10.1	10.41	1.49	6.27	2.47	50.39	26.17	180.09	223.70	133.29
57	59.49	10.25	31.41	1.00	8.5	8.50	1.43	8.05	2.46	191.25	39.03	369.60	253.32	181.87
58	52.73	7.22	35.53	1.39	9.9	7.12	1.75	8.90	2.81	124.22	59.63	260.62	293.28	207.39
59	55.97	5.82	38.14	1.16	9.0	7.76	1.55	8.65	3.51	138.50	48.37	227.79	258.55	217.02
60	54.24	10.22	32.40	1.17	9.6	8.21	1.58	8.28	2.67	104.99	39.03	253.22	269.28	207.51
61	56.99	7.68	31.50	1.35	9.2	6.81	1.69	9.64	3.06	101.11	20.00	254.14	283.99	219.36
62	54.01	6.65	35.78	1.27	8.5	6.69	1.60	9.24	3.46	119.90	52.33	237.68	308.25	197.67
63	67.4	5.31	44.31	0.81	10.0	12.35	1.43	9.39	2.98	137.36	39.12	190.76	272.32	204.56
64	71.36	12.41	51.15	0.90	10.9	12.11	1.51	6.19	1.47	140.53	37.93	175.14	222.02	135.59
65	67.93	9.87	47.57	0.70	9.5	13.57	1.38	5.82	1.53	87.91	28.25	171.62	201.00	149.83
66	59.39	4.79	39.94	1.02	8.1	7.94	1.42	5.77	1.83	61.57	39.11	135.40	241.32	136.22
67	72.67	4.99	47.78	0.76	10.0	13.16	1.42	9.67	2.54	103.74	41.48	196.37	290.91	239.75
68	69.02	7.20	51.49	0.74	9.8	13.24	1.40	11.16	2.87	121.72	56.90	225.05	312.14	281.37
69	64.81	7.04	45.78	1.09	9.1	8.35	1.51	7.51	2.02	123.00	10.56	207.23	233.04	170.10
70	65.54	3.12	45.47	0.96	7.9	8.23	1.37	8.81	3.24	100.67	41.27	225.83	282.81	213.27
71	56.98	9.49	39.28	0.85	9.7	11.41	1.42	11.25	2.81	145.25	42.82	227.85	260.06	275.78
72	58.19	8.50	43.68	1.12	9.0	8.04	1.52	10.34	3.57	213.10	40.37	235.10	274.35	213.25
73	67.57	5.22	48.22	1.23	9.1	7.40	1.60	10.66	3.21	106.40	44.63	213.33	282.29	211.82
74	61.57	9.42	41.37	0.93	9.2	9.89	1.42	7.31	1.24	94.87	34.67	210.12	186.44	157.98
75	69.68	9.56	48.21	0.97	10.0	10.31	1.49	9.80	3.18	122.51	58.24	180.83	273.81	205.79
76	68.26	11.34	49.75	1.12	9.9	8.84	1.56	8.26	2.20	119.03	34.14	228.57	223.66	167.43
77	71.50	9.70	47.50	1.37	10.0	7.30	1.73	7.25	2.22	134.17	23.99	210.14	239.58	151.08
78	60.47	5.39	41.05	1.50	9.1	6.07	1.80	8.43	2.21	108.70	36.44	192.67	258.67	184.27
79	70.31	10.80	49.52	1.14	10.0	8.77	1.58	8.15	2.27	152.52	41.57	328.95	230.28	176.55
80	70.33	8.62	47.84	1.11	10.0	9.01	1.56	8.30	2.17	106.50	22.61	279.93	209.23	163.32

Continuation Table S1.

Segregant	L	a	B	TA	SS	SAR	TI	TP*	TF*	TC*	TAC*	VC	FRAP*	ABTS*
81	52.73	15.50	26.06	1.42	10	7.04	1.77	7.78	2.28	140.77	56.27	250.01	246.93	163.81
82	74.56	5.56	44.32	1.07	9.4	8.79	1.51	8.31	2.64	91.77	21.94	197.37	229.03	173.41
83	71.07	9.14	48.86	1.07	9.3	8.69	1.50	10.59	3.25	130.51	25.12	269.80	328.83	223.03
84	65.66	7.77	44.92	0.71	7.0	9.86	1.20	16.59	6.40	151.59	46.41	251.66	361.70	312.30
85	71.19	5.24	44.52	1.02	9.6	9.41	1.49	9.49	2.71	112.85	29.93	227.55	269.63	218.74
86	72.13	4.66	43.59	0.95	9.1	9.58	1.43	9.57	2.77	94.74	20.97	174.38	274.17	213.98
87	59.04	5.18	40.18	0.80	8.2	10.25	1.31	8.73	3.20	77.36	37.07	165.00	326.22	191.25
88	71.74	9.37	50.40	0.92	8.2	8.91	1.37	9.49	2.29	135.33	35.10	321.44	273.79	211.58
89	71.64	8.67	47.36	1.42	9.1	6.41	1.74	5.36	1.91	114.49	26.24	165.32	223.59	166.38
90	64.14	10.46	40.02	1.02	10.0	9.80	1.51	8.09	2.29	181.86	44.80	232.50	227.63	175.12

L= brightness; a= coordinates red/green; b= coordinates yellow/blue; TA= titratable acidity (%); SS= soluble solids (°Brix); SAR= sugar/acidity ratio; TI= taste index; TP= total polyphenols (mg GAE g^{-1}); TF= total flavonoids (mg cat g^{-1}); TC= total carotenoids (μg β-carotene g^{-1}); TAC= total anthocyanin content (gly-3-glu 100 g^{-1}); VC= vitamin C (mg 100 g^{-1}); FRAP= antioxidant capacity FRAP method (μm Trolox g^{-1}); ABTS= antioxidant capacity ABTS method (μm Trolox g^{-1}); n.d.= not detected.
*Parameters are given per dry weight.

Table S2. Partial correlation between the chemical traits of tree tomato pulp. Bold means statistical significance ($p < 0.001$).

	TP	TF	FRAP	ABTS	TAC	TC	VC	TA	SS	SAR	TI
**TP**	1										
**TF**	**0,80**	1									
**FRAP**	-0,063	0,069	1								
**ABTS**	**0,534**	**-0,409**	**0,724**	1							
**TAC**	**0,329**	-0,042	0,166	-0,216	1						
**TC**	**0,471**	**-0,303**	**-0,306**	-0,112	-0,208	1					
**VC**	**0,496**	**-0,549**	0,010	-0,080	0,202	-0,027	1				
**TA**	0,124	-0,057	0,134	-0,093	-0,064	0,048	-0,220	1			
**SS**	0,247	-0,158	**-0,317**	-0,113	0,121	-0,072	-0,093	0,110	1		
**SAR**	0,119	-0,055	0,139	-0,088	-0,067	0,057	-0,222	**-0,998**	0,159	1	
**TI**	-0,134	0,062	-0,128	0,093	0,062	-0,040	0,232	**0,998**	-0,069	**0,993**	1

TP= total polyphenols; TF= total flavonoids; FRAP= antioxidant capacity FRAP method; ABTS= antioxidant capacity ABTS method; TC= total carotenoids; TAC= total anthocyanin content; VC= vitamin C; TA= titratable acidity; SS= soluble solids; SAR= sugar/acidity ratio; TI= taste index.
